# Supplementary material for: Overemphasis on publications may disadvantage historically excluded groups in STEM before and during COVID-19: A North American survey-based study
Source: PLoS One. 2023 Sep 27;18(9):e0291124. doi: 10.1371/journal.pone.0291124 (PMC10529568; doi:10.1371/journal.pone.0291124)
Supplement: S2 Fig — Smoothed density of A) first-authored and B) co-authored publications of respondents separated by career stage. Career stage grad represents graduate students, while postdoc represents postdoctoral scholars. (PDF) [file pone.0291124.s002.pdf]

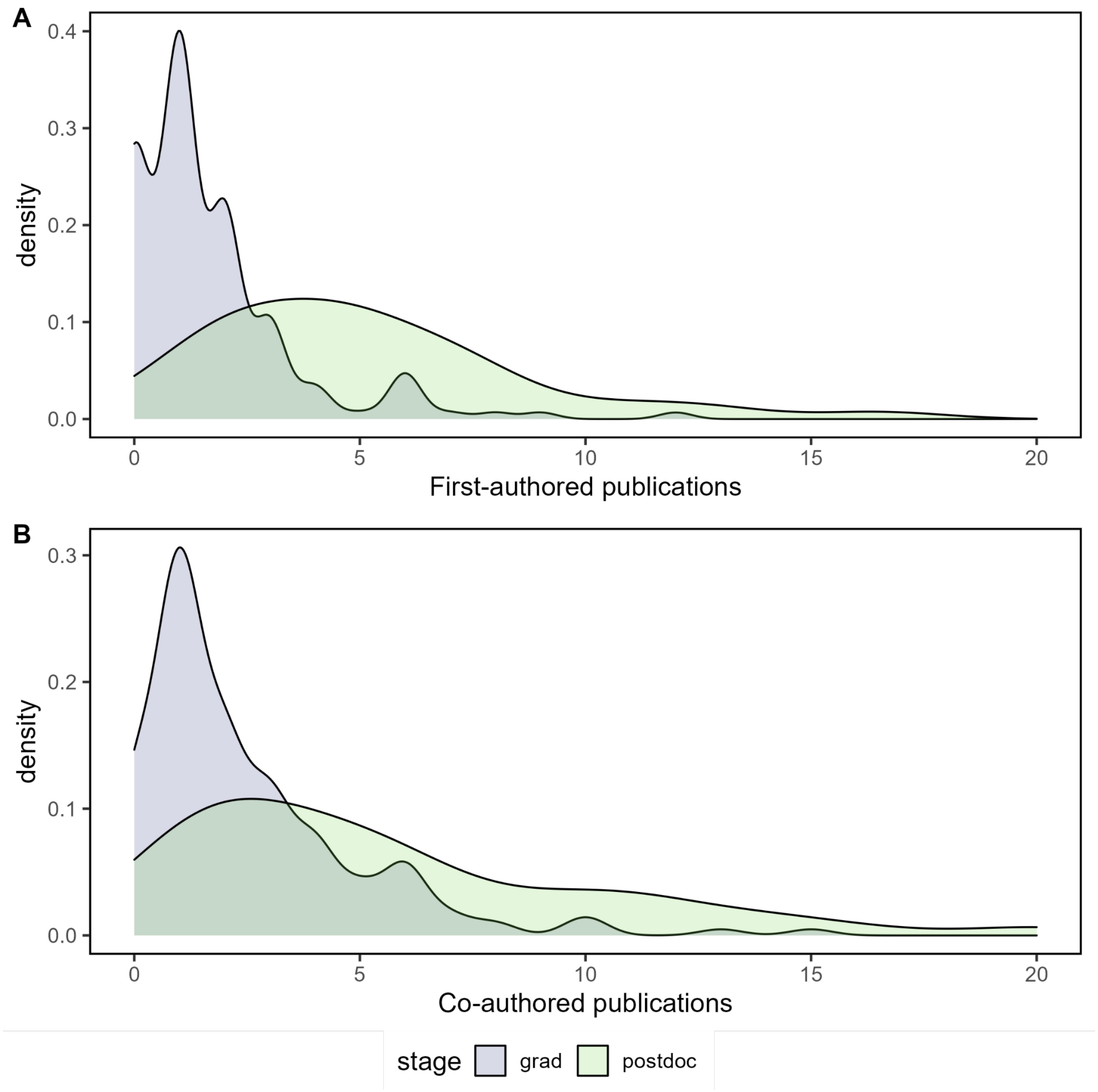

S2 Fig. Smoothed density of (A) first-authored and (B) co-authored publications of respondents separated by career stage. Career stage grad represents graduate students, while postdoc represents postdoctoral scholars.
