# Supplementary material for: Overemphasis on publications may disadvantage historically excluded groups in STEM before and during COVID-19: A North American survey-based study
Source: PLoS One. 2023 Sep 27;18(9):e0291124. doi: 10.1371/journal.pone.0291124 (PMC10529568; doi:10.1371/journal.pone.0291124)
Supplement: S1 Table — We allowed respondents to choose their identity preference or NA if they were uncomfortable answering. Below the results are reported as a percentage of total responses within each career stage. (PDF) [file pone.0291124.s003.pdf]

S1 Table. Breakdown of survey respondent identity. We allowed respondents to choose their identity preference or NA if they were uncomfortable answering. Below the results are reported as a percentage of total responses within each career stage.

	graduate students	postdoctoral scholars
**gender identity**		
male	24%	37%
female	73%	61%
non-binary or other	3%	3%
**BIPOC**		
yes	24%	9%
no	76%	91%
**first-generation college**		
yes	24%	24%
no	76%	76%
**chronic condition**		
yes	24%	9%
no	76%	89%
**English as a second language (ESL)**		
yes	18%	23%
no	82%	77%
