# Supplementary material for: Overemphasis on publications may disadvantage historically excluded groups in STEM before and during COVID-19: A North American survey-based study
Source: PLoS One. 2023 Sep 27;18(9):e0291124. doi: 10.1371/journal.pone.0291124 (PMC10529568; doi:10.1371/journal.pone.0291124)
Supplement: S2 Table — Except for years in graduate school, all other variables are factorial and coded as 1 = trainee identifies or 0 = trainee does not identify as first generation, female, BIPOC, having a chronic condition, or having English as a second language (ESL). Variables with 80% or higher probability of being on the same side of zero as the estimate (PD sign match) are bolded. 95% CRI = 95% credible interval, ESS = effective sample size. (PDF) [file pone.0291124.s004.pdf]

S2 Table. Graduate student results for Bayesian multiple regression of how years in graduate school and identity affect publication output. Except for years in graduate school, all other variables are factorial and coded as 1 = trainee identifies or 0 = trainee does not identify as first generation, female, BIPOC, having a chronic condition, or having English as a second language (ESL). Variables with 80% or higher probability of being on the same side of zero as the estimate (posterior distribution [PD] sign match) are bolded. 95% CRI = 95% credible interval, ESS = effective sample size.

**Parameter**	**Estimate (as median)**	**95% CRI**	**PD sign match**	R_{hat}	**ESS**
**Intercept**	1.22	[-0.05, 2.53]	99.9%	1.000	29,328
**Graduate training (yrs)**	0.52	[0.35, 0.68]	100%	1.000	22,914
First generation college	0.43	[-0.68, 1.57]	77.4%	1.000	22,783
Female	0.36	[-0.60, 1.29]	77.3%	1.000	20,581
BIPOC	-0.29	[-1.41, 0.80]	70.4%	1.000	24,615
**Chronic condition**	-0.78	[-1.98, 0.42]	90.1%	1.000	23,066
ESL	-0.22	[-1.23, 0.80]	66.5%	1.000	20,809
