# Supplementary material for: Overemphasis on publications may disadvantage historically excluded groups in STEM before and during COVID-19: A North American survey-based study
Source: PLoS One. 2023 Sep 27;18(9):e0291124. doi: 10.1371/journal.pone.0291124 (PMC10529568; doi:10.1371/journal.pone.0291124)
Supplement: S3 Table — Years spent as a graduate student and postdoctoral scholar are continuous, and all other variables are factorial and coded as 1 = trainee identifies or 0 = trainee does not identify as first generation, female, BIPOC, having a chronic condition, or having English as a second language (ESL). Variables with 80% or higher probability of being on the same side of zero as the estimate (PD sign match) are bolded. 95% CRI = 95% credible interval, ESS = effective sample size. (PDF) [file pone.0291124.s005.pdf]

S3 Table. **Postdoctoral scholar model results for Bayesian multiple regression of how years in training and identity affect publication output**. Years spent as a graduate student and postdoctoral scholar are continuous, and all other variables are factorial and coded as 1 = trainee identifies or 0 = trainee does not identify as first generation, female, BIPOC, having a chronic condition, or having English as a second language (ESL). Variables with 80% or higher probability of being on the same side of zero as the estimate (posterior distribution [PD] sign match) are bolded. 95% CRI = 95% credible interval, ESS = effective sample size.

**Parameter**	**Estimate (as median)**	**95% CRI**	**PD sign match**	**R_{hat}**	**ESS**
**Intercept**	5.95	[0.24, 11.61]	97.7%	1.000	18,033
**Graduate training (yrs)**	0.31	[-0.38, 1.02]	81.4%	1.000	19,649
**Postdoctoral training (yrs)**	2.10	[1.20, 2.82]	100%	1.000	18,911
First generation	-0.96	[-4.26, 2.37]	71.4%	1.000	19,174
**Female**	-2.47	[-5.32, 0.41]	95.4%	1.000	20,388
BIPOC	1.47	[-3.64, 6.61]	71.8%	1.000	18,320
**Chronic condition**	1.74	[-2.40, 5.81]	80.0%	1.000	22,130
ESL	-0.89	[-4.63, 2.78]	68.4%	1.000	17,862
