# Supplementary material for: Overemphasis on publications may disadvantage historically excluded groups in STEM before and during COVID-19: A North American survey-based study
Source: PLoS One. 2023 Sep 27;18(9):e0291124. doi: 10.1371/journal.pone.0291124 (PMC10529568; doi:10.1371/journal.pone.0291124)
Supplement: S4 Table — All estimates are in logit scale for ease of comparison. Variables with 80% or higher probability of being on the same side of zero as the estimate (PD sign match) are bolded. (PDF) [file pone.0291124.s006.pdf]

S4 Table. Results for binomial multiple regression on yes/no responses among graduate students to the question "*Has COVID-19 impacted your writing habits?*". All estimates are in logit scale for ease of comparison. Variables with 80% or higher probability of being on the same side of zero as the estimate (posterior distribution [PD] sign match) are bolded.

**Parameter**	**Estimate (as median)**	**95% CRI**	**PD sign match**	**R_{hat}**	**ESS**
Intercept	-0.32	[-1.09, 0.45]	78.8%	1.000	26,253
Graduate training (yrs)	0.02	[-0.10, 0.15]	63.4%	1.000	22,234
**First generation**	0.90	[0.04, 1.86]	98.0%	1.000	21,497
**Female**	1.21	[0.55, 1.88]	99.9%	1.000	21,519
**BIPOC**	1.97	[0.98, 3.16]	100%	1.000	18,796
**Chronic condition**	0.48	[-0.43, 1.48]	84.8%	1.000	19,856
**ESL**	-1.12	[-1.87, -0.38]	99.9%	1.000	19,640
