# Supplementary material for: Overemphasis on publications may disadvantage historically excluded groups in STEM before and during COVID-19: A North American survey-based study
Source: PLoS One. 2023 Sep 27;18(9):e0291124. doi: 10.1371/journal.pone.0291124 (PMC10529568; doi:10.1371/journal.pone.0291124)
Supplement: S5 Table — All estimates are in logit scale for ease of comparison. Variables with 85% or higher probability of being on the same side of zero as the estimate (PD sign match) are bolded. (PDF) [file pone.0291124.s007.pdf]

S5 Table. Binomial multiple regression on yes/no responses among postdoctoral scholars to the question "*Has COVID-19 impacted your writing habits?*". All estimates are in logit scale for ease of comparison. Variables with 85% or higher probability of being on the same side of zero as the estimate (posterior distribution [PD] sign match) are bolded.

**Parameter**	**Estimate (as median)**	**95% CRI**	**PD sign match**	**R_{hat}**	**ESS**
Intercept	-0.33	[-1.43, 0.73]	72.8%	1.000	20,477
**Postdoc training (yrs)**	0.29	[-0.01, 0.62]	97.1%	1.000	16,559
First generation	0.48	[-0.73, 1.76]	77.7%	1.000	16,298
**Female**	0.90	[-0.13, 2.00]	95.7%	1.000	18,330
BIPOC	0.43	[-1.45, 2.44]	67.5%	1.000	18,174
**Chronic condition**	-1.31	[-2.96, 0.21]	95.3%	1.000	20,196
**ESL**	-1.28	[-2.74, 0.08]	96.8%	1.000	14,096
