# Supplementary material for: Overemphasis on publications may disadvantage historically excluded groups in STEM before and during COVID-19: A North American survey-based study
Source: PLoS One. 2023 Sep 27;18(9):e0291124. doi: 10.1371/journal.pone.0291124 (PMC10529568; doi:10.1371/journal.pone.0291124)
Supplement: S6 Table — They generally reported having less productivity and less motivation. (PDF) [file pone.0291124.s008.pdf]

S6 Table. Five of eight non-binary/other gendered respondents indicated that the COVID-19 pandemic impacted writing habits. They generally reported having less productivity and less motivation.

**Question**	**Much less**	**Less**	**Neutral/ no change**	**More**	**Much more**
Amount of time for writing	1	0	1	3	0
Writing productivity	0	5	0	0	0
Writing motivation	3	1	1	0	0
