# Supplementary material for: Overemphasis on publications may disadvantage historically excluded groups in STEM before and during COVID-19: A North American survey-based study
Source: PLoS One. 2023 Sep 27;18(9):e0291124. doi: 10.1371/journal.pone.0291124 (PMC10529568; doi:10.1371/journal.pone.0291124)
Supplement: S1 File — This copy shows exact question phrasing and survey structure. (PDF) [file pone.0291124.s009.pdf]

Trainee attitudes toward scientific writing

Start of Block: Disclosure statement

Welcome to our survey on trainee attitudes toward scientific writing!
We are a group of graduate students and postdocs from Yale University. We are conducting a research study to examine environmental biology trainee challenges and attitudes when it comes to academic writing, and potential writing support strategies to make publishing easier. Participation in this study will involve completing a survey and will take approximately 9 minutes.
There are no known or anticipated risks to you for participating. Although this study will not benefit you personally, our results will add to the knowledge about barriers to academic writing. We will publish our results in a peer-reviewed journal, where we hope that our results will help other trainees reach their writing goals.
All of your responses will be anonymous. Only the researchers involved in this study and those responsible for research oversight will have access to the information you provide. At no point will we ask you for your name or email address. There is an option at the end of the survey to enter a drawing for a $20 giftcard to bookshop.org. This information is not linked with your survey responses.
Participation in this study is completely voluntary. You are free to decline to participate, to end participation at any time for any reason, or to refuse to answer any individual question. If you have any questions about this study, you may contact one of the investigators (Freya Rowland [freya.rowland@yale.edu], Yara Alshwairihk [yara.alshwairikh@yale.edu], Ana Fanton [ana.fantonborges@yale.edu], Mary Burak [mary.burak@yale.edu], Kyra Prats [kyra.prats@yale.edu], or Marlyse Duguid [marlyse.duguid@yale.edu]).
If you would like to talk with someone other than the researchers to discuss problems or concerns, to discuss situations in the event that a member of the research team is not available, or to discuss your rights as a research participant, you may contact the Yale University Human Subjects Committee, 203-785-4688, human.subjects@yale.edu. Additional information is available at https://your.yale.edu/research-support/human-research/research-participants/rights-research-participant
Would you like to participate in the study?

○ Yes (1)

○ No (2)

Skip To: End of Survey If Welcome to our survey on trainee attitudes toward scientific writing! We are a group of graduate... = No

End of Block: Disclosure statement

Start of Block: Trainee status

Are you a graduate student or postdoctoral associate/fellow?

○ Yes (1)

○ No (2)

Skip To: End of Survey If Are you a graduate student or postdoctoral associate/fellow? = No

What is your subfield of biological/environmental sciences?

____

How many cumulative years have you spent so far/did you spend in graduate school (include all degrees)?

	0 1 2 3 4 5 6 7 8 9 10 11 12 13 14 15
Years of graduate study ()	

How many cumulative years have you spent so far as a postdoctoral associate/fellow?

	Not Applicable<br>0 1 2 3 4 5 6 7 8 9 10
Years as postdoc ()	

Please rank career goals post-training from 0-10. 0 = no interest, 10 = most interest. You may use numbers more than once.

______ Academic teaching-focused (e.g, community college, liberal arts college) (1)
______ Academic teaching and research (e.g., R2, R3, master's granting universities) (5)
______ Academic research-intensive (R1) (6)
______ Government research and/or monitoring (local, state, or federal) (7)
______ Data science and/or industry (10)
______ Outreach/science communication (11)
______ Non-governmental organization (NGO) (14)
______ Unsure (12)

End of Block: Trainee status

Start of Block: Writing experience

Did you publish any peer-reviewed scientific papers as first or co-author prior to starting your graduate work?

○ Yes (1)

○ No (2)

How many accepted or published peer-reviewed scientific papers do you currently have as **first-author**?

	0	2	4	6	8	10	12	14	16	18	20
Total first-author publications ()											

How many accepted or published peer-reviewed scientific papers do you currently have as **co-author** (non-first author)?

	0	2	4	6	8	10	12	14	16	18	20
Total co-authored publications ()											

Have you published one or more peer-reviewed scientific papers **without an advisor/PI** on the author list?

◯ Yes (2)

◯ No (3)

End of Block: Writing experience

Start of Block: Lab Group or PI involvement in writing

Do your lab members provide feedback on your writing?

◯ Yes (1)

◯ No (2)

Display This Question:

If Do your lab members provide feedback on your writing? = Yes

Has lab feedback improved your writing skills?

◯ The feedback has helped (1)

◯ The feedback has not helped (2)

Have you collaborated on papers and/or grants with your lab members?

◯ Yes (1)

◯ No (2)

How is your advisor/PI involved in your writing? (Check all that apply)

- ▢ Planning (generating ideas) (1)
- ▢ Drafting or outlining (2)
- ▢ Writing sections of manuscript (3)
- ▢ Revising with many suggestions and comments (4)
- ▢ Revising with minor comments (5)
- ▢ My advisor is not involved in my writing (6)

In your current position how many revisions do you go through with your PI before submission?

- ○ 1 (1)
- ○ 1-3 (2)
- ○ 3-5 (3)
- ○ 5+ (4)

End of Block: Lab Group or PI involvement in writing

Start of Block: Writing habits

Generally, how many hours per week do you write, edit, and revise manuscripts?

	0	3	6	9	12	15	18	21	24	27	30
Hours per week ()											

How often do you write? (Check all that apply)

- ☐ I set a specific amount of time each week (e.g., 15 minutes per day, 2 hours on Tuesday/Thursday) (1)
- ☐ I set aside specific days each week (2)
- ☐ I set aside large blocks of time (e.g., during weekends or breaks) (8)
- ☐ I set aside large blocks of time before deadlines (3)
- ☐ I do not track or schedule my writing time (6)

Where do you usually write (pre-COVID)? (Check all that apply)

- ☐ Home (off-campus residence or dormitory) (1)
- ☐ University provided office or lab space (2)
- ☐ On-campus spaces (e.g. graduate student centers, affinity group centers) (3)
- ☐ University or public libraries (4)
- ☐ Coffee shops/restaurants (5)
- ☐ Other (please specify) (6) ____
- ☐ I would like to write at an office, but my university does not provide me with office space (8)

How do you track your writing progress? (Check all that apply)

- ▢ Electronic spreadsheets (1)
- ▢ Electronic note taking applications (2)
- ▢ Physical notebook (3)
- ▢ Checking in with writing accountability/support group (4)
- ▢ Checking in with advisor or mentor (5)
- ▢ I do not track my writing progress (6)
- ▢ Other (please specify) (7) ____

End of Block: Writing habits

Start of Block: Writing Groups

Have you ever participated in a writing support group (e.g., writing workshop, course, and/or peer group) during your graduate or post-graduate work?

- ○ Yes (1)
- ○ No (2)

Display This Question:

If Have you ever participated in a writing support group (e.g., writing workshop, course, and/or pee... = No

Why did you not participate in a writing support group? (Check all that apply)

- ☐ I could not find a group to join (2)
- ☐ I had no interest in joining a writing group (4)
- ☐ I had ample writing support within my immediate graduate group (e.g. advisor, laboratory) (5)
- ☐ I did not know writing support groups existed (6)

Display This Question:

If Have you ever participated in a writing support group (e.g., writing workshop, course, and/or pee... = Yes

Please mark which type(s) of writing support groups you participated in. (Check all that apply)

- ☐ Formal writing course (1)
- ☐ Formal writing workshop (3)
- ☐ Peer group (5)
- ☐ Mentoring program (6)
- ☐ Writing retreats, study halls, etc. (8)

Display This Question:

If Have you ever participated in a writing support group (e.g., writing workshop, course, and/or pee... = Yes

How did your perspective on writing change through participating in a writing group?

- I like writing less (1)
- Neutral/no change (2)
- I like writing more (3)

Display This Question:

If Have you ever participated in a writing support group (e.g., writing workshop, course, and/or pee... = Yes

What aspects of writing were addressed within the writing support group and to what extent was this beneficial?

	Worsened (1)	No Change (2)	Improved (3)	Not Applicable (4)
Goal setting (1)	○	○	○	○
Giving and/or receiving reviews (2)	○	○	○	○
Collaboration (4)	○	○	○	○
Camaraderie (5)	○	○	○	○
Technical writing skills (6)	○	○	○	○
Less difficulty starting a writing project (8)	○	○	○	○
Time management (9)	○	○	○	○
Overcoming "perfectionism paralysis" (10)	○	○	○	○
Overcoming writing anxiety (18)	○	○	○	○
Overcoming imposter syndrome (19)	○	○	○	○
Writing quality (20)	○	○	○	○
Writing output (21)	○	○	○	○

End of Block: Writing Groups

Start of Block: Writing Challenges

What are some of the challenges you face with writing (pre-COVID-19)? (Check all that apply)

- ▢ I find it difficult to start a new writing project (1)
- ▢ I have trouble fitting writing into my schedule (2)
- ▢ I have too many other obligations (3)
- ▢ I do not receive adequate feedback on my writing (4)
- ▢ My perfectionism with writing hinders my progress (5)
- ▢ I get easily distracted whenever I try to write (6)

End of Block: Writing Challenges

Start of Block: Impact of the COVID-19 pandemic on writing habits

Has the COVID-19 pandemic impacted your writing habits?

- ○ Yes (1)
- ○ No (2)

Skip To: End of Block If Has the COVID-19 pandemic impacted your writing habits? = No

Has the pandemic impacted the amount of time you have for writing?

- ◯ I have much less time for writing (1)
- ◯ I have less time for writing (2)
- ◯ Neutral/no change (3)
- ◯ I have more time for writing (6)
- ◯ I have much more time for writing (7)

Has the pandemic affected your writing productivity?

- ◯ I am much less productive (1)
- ◯ I am less productive (2)
- ◯ Neutral/no change (3)
- ◯ I am more productive (4)
- ◯ I am much more productive (5)

Has the pandemic impacted your motivation to write?

- ◯ I am much less motivated to write (1)
- ◯ I am less motivated to write (2)
- ◯ Neutral/no change (3)
- ◯ I am more motivated to write (4)
- ◯ I am much more motivated to write (5)

Has the pandemic affected your participation in writing support groups?

○ I participate much less (1)

○ I participate less (2)

○ Neutral/no change (3)

○ I participate more (5)

○ I participate much more (6)

○ I have not participated or am not currently in a writing support group (7)

End of Block: Impact of the COVID-19 pandemic on writing habits

---

Start of Block: Writing attitudes

Please use one word to describe your overall feelings about the scientific writing process:

○ 3 (3) ____

Please use one word to describe your overall feelings about the peer-review process:

○ 6 (6) ____

End of Block: Writing attitudes

Start of Block: Demographic information

Are you currently and/or were you previously at a U.S. or Canadian university/institution?

○ Yes (1)

○ No (2)

Which of the following most closely aligns with your gender identity?

- ◯ Female (1)
- ◯ Male (2)
- ◯ Non-binary / third gender (3)
- ◯ Prefer to self-identify: (5) ____
- ◯ Prefer not to say (4)

Is English your primary and/or first language?

- ◯ Yes (1)
- ◯ No (2)
- ◯ Other (specify): (3) ____

Are you the first in your family to obtain a college degree?

- ◯ Yes (1)
- ◯ No (2)
- ◯ Prefer not to answer (3)

Do you identify as Black, Indigenous, and/or as a person of color?

○ Yes (1)

○ No (2)

○ Prefer not to answer (3)

Do you have a disability or chronic health condition?

○ Yes (1)

○ No (2)

○ Prefer not to answer (3)

End of Block: Demographic information

Start of Block: Open response

Is there anything else you'd like to add about your writing experiences? (Optional)

____

End of Block: Open response

Start of Block: Redirect to gift card

Thank you for taking the survey! Would you like to enter a drawing for a $20 gift card to bookshop.org?

○ Yes (1)

○ No (2)

End of Block: Redirect to gift card
