# Supplementary material for: Overemphasis on publications may disadvantage historically excluded groups in STEM before and during COVID-19: A North American survey-based study
Source: PLoS One. 2023 Sep 27;18(9):e0291124. doi: 10.1371/journal.pone.0291124 (PMC10529568; doi:10.1371/journal.pone.0291124)
Supplement: S1 Fig — Bigger words indicate higher occurrence in the survey responses. (PDF) [file pone.0291124.s001.pdf]

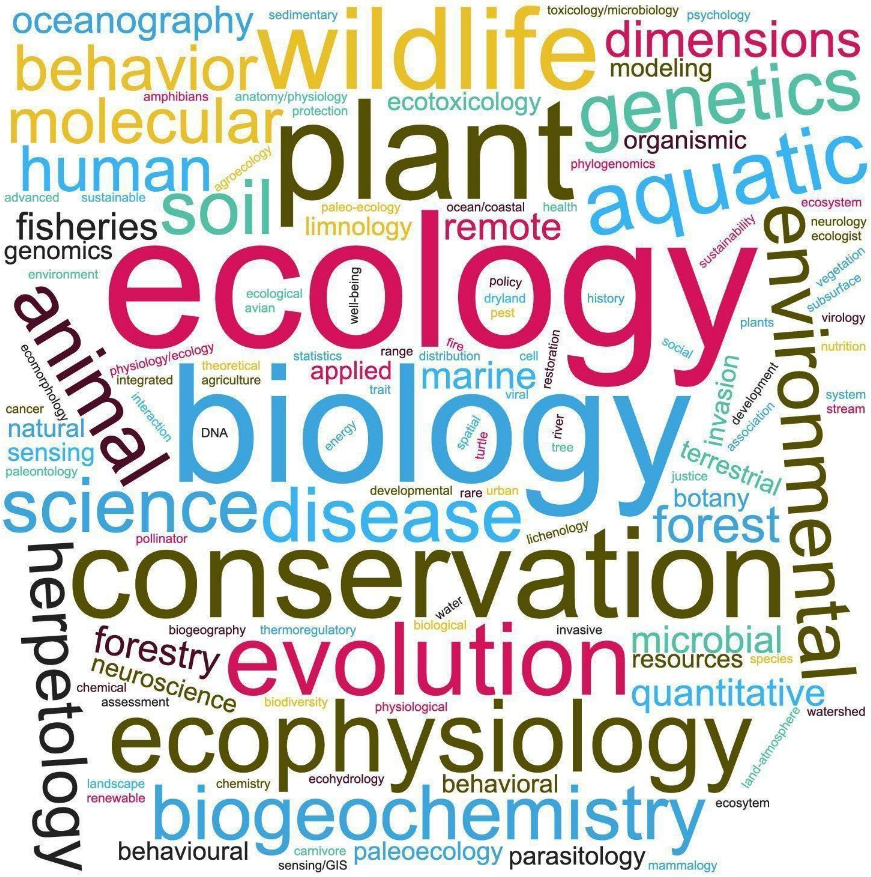

S1 Fig. Word cloud representation of respondents' subfield of environmental biology. Bigger words indicate higher occurrence in the survey responses.
