# Supplementary material for: Overemphasis on publications may disadvantage historically excluded groups in STEM before and during COVID-19: A North American survey-based study
Source: PLoS One. 2023 Sep 27;18(9):e0291124. doi: 10.1371/journal.pone.0291124 (PMC10529568; doi:10.1371/journal.pone.0291124)
Supplement: Attachment — Submitted filename: PLOS R3 response.docx [file pone.0291124.s010.docx]

17 August 2023

Dear Dr. Safayi,

We thank you for your quick and thoughtful review of our manuscript (PONE-D-22-24997R2). We have incorporated all of the changes both you and the reviewer suggested and think it clarified and strengthened our manuscript.

Below we detail point-by-point responses to the comments.

We look forward to having this paper published in PLOS ONE. Thank you again for the work you put in to helping us get this across the finish line.

Best regards,

Freya Rowland and Kyra Prats, on behalf of all coauthors

—----

PONE-D-22-24997R2

Overemphasis on publications may disadvantage historically excluded groups in STEM before and during COVID-19: a survey-based study

PLOS ONE

Dear Dr. Rowland,

Thank you for submitting your manuscript to PLOS ONE. After careful consideration, we feel that it has merit but does not fully meet PLOS ONE’s publication criteria as it currently stands. Therefore, we invite you to submit a revised version of the manuscript that addresses the points raised during the review process.

You have done a great job in concluding your project. However, I'd like you to please:

1. Add a section where you address "limitations of the Study" where you can use some of the responses you've written for the previous reviewers. I'd also like you to share your suggestions of improvements if you or anyone else wants to repeat this study or conclude a similar one.

**We have added a section titled “limitations of the study” to the Discussion to expand upon what we wish we could have measured but didn’t. This expanded our paragraph about intersectionality:**

**“Given differences in publishing and research between STEM fields, our results most prominently reflect the experiences of environmental biology trainees; while our findings can inform our understanding of the pandemic’s impact across STEM field, future research is needed to explicitly disentangle the effects of the pandemic on identity in a wide range of STEM fields.** *We chose an anonymous survey mechanism to maximize and standardize our sample size, but in doing so, we lack the nuance we could have included if our study had also allowed for follow-up interviews. The survey questions were subject to interpretation and reporting differences among respondents. Because of this, our analyses had to be neutral; for example, we asked trainees whether the pandemic impacted their writing, however we cannot fully conclude it was a negative impact, despite follow-up questions showing a perceived lack of motivation and productivity. Additionally, although ESL trainees did not report effects of the pandemic on writing habits, it could very well be that cultural stigmas around discussing difficulties prevented ESL and international students from answering our questions honestly, especially considering the free responses indicated writing was much more challenging for these trainees (Table 1). The survey also lacked the ability to differentiate between ESL trainees who reside in their home country and international students. Furthermore, caretaker status would have been an informative metric due to school and care center closure during the pandemic, as this likely had a very strong influence on productivity. Future research should disentangle these effects further.*”

2. You briefly address ESL in your supporting materials. I'd like you to elaborate on ESL and internationals with one or two paragraph in your main text. Over one third of US trainees are internationals. They deal with other marginalizing factors on top of what US-born students do. "Accent Trumps Race" for example. Some come from the "Global South." And, as the last reviewer mentioned, they might hesitate to share their thoughts in a survey because of the cultural differences and hesitations. In the pandemic, and despite the privilege that American students had, many internationals didn't get the chance to see their families, were in exile, and that cannot be ignored. I do not expect you to spend more than a couple of paragraphs on this. I just want you to acknowledge their hardship. You can elaborate on this in the discussion or in the "limitations of the study" for example.

**We agree that the ESL results were compelling, and that is a large part of the reason we included the free responses as a table in the main paper (Table 1). Although the two first-authors are from the U.S. with English as a first language, we’d like to note that two of the co-authors are international trainees with ESL. We have added more to the discussion about the experience of ESL trainees.**

**We didn’t want to speculate too much about the experiences of trainees, but we agree that highlighting the COVID experience of international students is important. We have added a sentence to the discussion: “***In the case of trainees with ESL – many of whom are likely international trainees – the pandemic prevented many from being able to see their families. However, these trainees already were physically distanced from their families even prior to the pandemic, which could explain why they were less likely to report the pandemic affected their writing habits given that they could have already been experiencing isolation*.**”**

**We also have a paragraph about ESL trainees in the discussion highlighting how much harder writing is in general:**

*“Trainees with ESL had slightly fewer publications than trainees with English as a first language. Although these trends were weak, many respondents explicitly mentioned the struggles of English as the default language of science in the open response section (Table 1). Research shows that trainees with ESL face enormous obstacles to scientific publishing [35] but writing intervention programs have helped this group gain confidence and skills in scientific writing [8]. Universities should invest in extra resources for trainees with ESL, including discipline-specific writing courses [36,37].”*

**Finally, we discuss the ways in which we could have expanded on the survey questions related to the ESL experience in our new “Limitations of the study” section:**

*“Additionally, although ESL trainees did not report effects of the pandemic on writing habits, it could very well be that cultural stigmas around discussing difficulties prevented ESL trainees from answering our questions honestly, especially considering the free responses indicated writing in general was much more challenging for these trainees (Table 1). The survey also lacked the ability to differentiate between ESL trainees who reside in their home country and international students.”*

Minor revisions:

- Title: Add “in the United States” at the end of the title. The word “historically” imposes a different meaning to the word “disadvantaged” globally. As a reminder, PLOS ONE is a global platform with readers and authors from across the world.

**We have edited the title to say “North America” instead of the United States because we sent targeted emails to both U.S. and Canadian institutions. However, it is important to note that we likely had responses from worldwide. Although we did not track locations of responses in our main survey, the option gift card drawing indicated responses from Europe and Australia as well.**

- Introduction > Line 3: “hereafter, postdocs” change to “hereafter, trainees”

**Edited.**

I understand that the COVID is almost over, and you might think (as you mentioned in an email to me) that the readers might not find this as relevant. I respectfully disagree. You did such a great job in addressing one of the least acknowledged topics. The impact of the COVID is just an example of each intervention for the marginalized/excluded groups you targeted. It will be as relevant as any imposed intervention. To save you time, and as soon as I receive a new revision submission, I will accept it without sending it for review again. In other words, consider this "minor revision" as a "conditionally accepted" letter from me. I look forward to receiving your resubmission.

**Thank you for expediting our manuscript. The timeliness is a top concern of the authors and we are relieved to hear you believe it is still relevant.**

Please submit your revised manuscript by Sep 29 2023 11:59PM. If you will need more time than this to complete your revisions, please reply to this message or contact the journal office at plosone@plos.org. When you're ready to submit your revision, log on to <https://www.editorialmanager.com/pone/> and select the 'Submissions Needing Revision' folder to locate your manuscript file.

Please include the following items when submitting your revised manuscript:

- A rebuttal letter that responds to each point raised by the academic editor and reviewer(s). You should upload this letter as a separate file labeled 'Response to Reviewers'.
- A marked-up copy of your manuscript that highlights changes made to the original version. You should upload this as a separate file labeled 'Revised Manuscript with Track Changes'.
- An unmarked version of your revised paper without tracked changes. You should upload this as a separate file labeled 'Manuscript'.

If you would like to make changes to your financial disclosure, please include your updated statement in your cover letter. Guidelines for resubmitting your figure files are available below the reviewer comments at the end of this letter.

If applicable, we recommend that you deposit your laboratory protocols in [protocols.io](http://protocols.io/) to enhance the reproducibility of your results. Protocols.io assigns your protocol its own identifier (DOI) so that it can be cited independently in the future. For instructions see: <https://journals.plos.org/plosone/s/submission-guidelines#loc-laboratory-protocols>. Additionally, PLOS ONE offers an option for publishing peer-reviewed Lab Protocol articles, which describe protocols hosted on [protocols.io](http://protocols.io/). Read more information on sharing protocols at <https://plos.org/protocols?utm_medium=editorial-email&utm_source=authorletters&utm_campaign=protocols>.

We look forward to receiving your revised manuscript.

Kind regards,

Sina Safayi, D.V.M., Ph.D.
Academic Editor
PLOS ONE

Journal Requirements:

Please review your reference list to ensure that it is complete and correct. If you have cited papers that have been retracted, please include the rationale for doing so in the manuscript text, or remove these references and replace them with relevant current references. Any changes to the reference list should be mentioned in the rebuttal letter that accompanies your revised manuscript. If you need to cite a retracted article, indicate the article’s retracted status in the References list and also include a citation and full reference for the retraction notice.

[Note: HTML markup is below. Please do not edit.]

Reviewers' comments:

Reviewer's Responses to Questions

**Comments to the Author**

1. If the authors have adequately addressed your comments raised in a previous round of review and you feel that this manuscript is now acceptable for publication, you may indicate that here to bypass the “Comments to the Author” section, enter your conflict of interest statement in the “Confidential to Editor” section, and submit your "Accept" recommendation.

Reviewer #4: (No Response)

2. Is the manuscript technically sound, and do the data support the conclusions?

The manuscript must describe a technically sound piece of scientific research with data that supports the conclusions. Experiments must have been conducted rigorously, with appropriate controls, replication, and sample sizes. The conclusions must be drawn appropriately based on the data presented.

Reviewer #4: Yes

3. Has the statistical analysis been performed appropriately and rigorously?

Reviewer #4: I Don't Know

4. Have the authors made all data underlying the findings in their manuscript fully available?

The [PLOS Data policy](http://www.plosone.org/static/policies.action#sharing) requires authors to make all data underlying the findings described in their manuscript fully available without restriction, with rare exception (please refer to the Data Availability Statement in the manuscript PDF file). The data should be provided as part of the manuscript or its supporting information, or deposited to a public repository. For example, in addition to summary statistics, the data points behind means, medians and variance measures should be available. If there are restrictions on publicly sharing data—e.g. participant privacy or use of data from a third party—those must be specified.

Reviewer #4: Yes

5. Is the manuscript presented in an intelligible fashion and written in standard English?

PLOS ONE does not copyedit accepted manuscripts, so the language in submitted articles must be clear, correct, and unambiguous. Any typographical or grammatical errors should be corrected at revision, so please note any specific errors here.

Reviewer #4: Yes

6. Review Comments to the Author

Please use the space provided to explain your answers to the questions above. You may also include additional comments for the author, including concerns about dual publication, research ethics, or publication ethics. (Please upload your review as an attachment if it exceeds 20,000 characters)

Reviewer #4: I recommend accepting this manuscript.
The topic and research goals of the “Overemphasis on publications may disadvantage historically excluded groups in STEM before and during COVID-19: a survey-based study” is very relevant. Moreover, The authors have adequately addressed reviewers comments.

I hope their major observation that negative impact of pandemic on writing habits among BIPOC and female graduate students and postdocs inspires discussion and action within higher ed institutions. The compounding effects of social isolation, lack of peer networks and advisor mentorship, institutional writing support, mental health decline and community violence and harm presented during the pandemic can’t be brushed aside as we go into ‘business as usual mode’ in academia. I support the authors call for inclusive metrics for new faculty hiring that doesn’t prioritize publications above all else.

**Thank you! This means a lot that you connected with the message of our manuscript.**

Besides, the central observation, the following results also piqued my interest:
• 40% of graduate students had published before starting graduate school- I wonder if this is standard for STEM disciplines now or an outlier for environmental sciences. This would further create disparity among undergraduates (predominantly historically excluded groups) who don’t have early access to research opportunities.

**That is a good point. Many people from HEGs would not likely have access to research opportunities or the support necessary to publish. A publication is certainly helpful for getting into top graduate programs - it would be really interesting to see how pre-graduate school publications affect admissions at top schools and access to scholarship and fellowship opportunities. Unfortunately, we don’t have any location information for our respondents.**

• While ESL students were the only population to provide text responses on challenges, they weren’t forthcoming on communicating lack of motivation due to pandemic. What percent of these ESL students are international? I wonder if this is a consequence of cultural stigma around reporting mental health decline. I also wonder whether Generative AI tool or writing assistant apps can mitigate writing challenges for ESL groups.

**We agree that this is interesting. The survey was anonymous, but it could very well be that cultural stigmas around reporting difficulties prevented ESL and international students from answering our questions honestly. We have added some of this to the discussion: “***We chose an anonymous survey mechanism to maximize and standardize our sample size, but in doing so, we lack the nuance we could have included if our study had also allowed for follow-up interviews. The survey questions were subject to interpretation and reporting differences among respondents. Because of this, our analyses had to be neutral – e.g., we asked trainees whether the pandemic impacted their writing, however we cannot conclude it was a negative impact. Additionally, although ESL trainees did not report effects of the pandemic on writing habits, it could very well be that cultural stigmas around discussing difficulties prevented ESL and international students from answering our questions honestly – especially considering the free responses indicated writing was much more challenging for these trainees (Table 1). The survey also lacked the ability to differentiate between ESL trainees who reside in their home country and international students.***”**

**Regarding the proportion of ESL students that are international, that was a discussion we had early on. Being an international student in no way dictates that you are communicating in a second or third language (e.g., a Canadian or UK resident in the U.S.), and ESL students can also be from the same country but have parents with a different primary language. Unfortunately our survey is not able to tease this apart, but there are a large portion of Americans that come from Spanish-speaking households, for example, so we chose to leave it as ESL but added a sentence to the discussion (see above) about how we were unable to separate ESL and international trainees, but it would be very useful for future studies.**

**Interesting point about AI perhaps being helpful for ESL trainees. We lack any sort of data currently on the directions of AI and how it will be incorporated into academic writing, but hopefully it is useful and makes the process a bit easier on those whose first language is not English.**

7. PLOS authors have the option to publish the peer review history of their article ([what does this mean?](https://journals.plos.org/plosone/s/editorial-and-peer-review-process#loc-peer-review-history)). If published, this will include your full peer review and any attached files.


If you choose “no”, your identity will remain anonymous but your review may still be made public.


**Do you want your identity to be public for this peer review?** For information about this choice, including consent withdrawal, please see our [Privacy Policy](https://www.plos.org/privacy-policy).

Reviewer #4: No

[NOTE: If reviewer comments were submitted as an attachment file, they will be attached to this email and accessible via the submission site. Please log into your account, locate the manuscript record, and check for the action link "View Attachments". If this link does not appear, there are no attachment files.]

While revising your submission, please upload your figure files to the Preflight Analysis and Conversion Engine (PACE) digital diagnostic tool, <https://pacev2.apexcovantage.com/>. PACE helps ensure that figures meet PLOS requirements. To use PACE, you must first register as a user. Registration is free. Then, login and navigate to the UPLOAD tab, where you will find detailed instructions on how to use the tool. If you encounter any issues or have any questions when using PACE, please email PLOS at figures@plos.org. Please note that Supporting Information files do not need this step.
